# Tissue-Specific Increases in 11β-Hydroxysteroid Dehydrogenase Type 1 in Normal Weight Postmenopausal Women

**DOI:** 10.1371/journal.pone.0008475

**Published:** 2009-12-29

**Authors:** Therése Andersson, Kotryna Simonyte, Ruth Andrew, Magnus Strand, Jonas Burén, Brian R. Walker, Cecilia Mattsson, Tommy Olsson

**Affiliations:** 1 Division of Medicine, Department of Public Health and Clinical Medicine, Umeå University Hospital, Umeå, Sweden; 2 Endocrinology Unit, Centre for Cardiovascular Science, Queen's Medical Research Institute, University of Edinburgh, Edinburgh, United Kingdom; University of Tor Vergata, Italy

## Abstract

With age and menopause there is a shift in adipose distribution from gluteo-femoral to abdominal depots in women. Associated with this redistribution of fat are increased risks of type 2 diabetes and cardiovascular disease. Glucocorticoids influence body composition, and 11β-hydroxysteroid dehydrogenase type 1 (11βHSD1) which converts inert cortisone to active cortisol is a putative key mediator of metabolic complications in obesity. Increased 11βHSD1 in adipose tissue may contribute to postmenopausal central obesity. We hypothesized that tissue-specific 11βHSD1 gene expression and activity are up-regulated in the older, postmenopausal women compared to young, premenopausal women. Twenty-three pre- and 23 postmenopausal, healthy, normal weight women were recruited. The participants underwent a urine collection, a subcutaneous adipose tissue biopsy and the hepatic 11βHSD1 activity was estimated by the serum cortisol response after an oral dose of cortisone. Urinary (5α-tetrahydrocortisol+5β-tetrahydrocortisol)/tetrahydrocortisone ratios were higher in postmenopausal women versus premenopausal women in luteal phase (*P*<0.05), indicating an increased whole-body 11βHSD1 activity. Postmenopausal women had higher 11βHSD1 gene expression in subcutaneous fat (*P*<0.05). Hepatic first pass conversion of oral cortisone to cortisol was also increased in postmenopausal women versus premenopausal women in follicular phase of the menstrual cycle (*P*<0.01, at 30 min post cortisone ingestion), suggesting higher hepatic 11βHSD1 activity. In conclusion, our results indicate that postmenopausal normal weight women have increased 11βHSD1 activity in adipose tissue and liver. This may contribute to metabolic dysfunctions with menopause and ageing in women.

## Introduction

With age, and menopause, in women there is a shift in adipose distribution from gluteo-femoral to abdominal depots [1], [2]. Abdominal fat accumulation, and menopause per se, links to an increased risk of insulin resistance, type 2 diabetes, and future cardiovascular disease [3], [4]. The decrease in estradiol (E2) levels that occur with menopause might be an underlying factor of these dysmetabolic features [5]. However, other steroid hormones, notably glucocorticoids, may directly or indirectly influence body composition and the risk of cardiovascular disease [6].

Circulating cortisol levels are tightly controlled by forward drive through hypothalamic-pituitary factors and negative feedback by cortisol[7]. Glucocorticoid excess due to tumours producing ACTH or cortisol, as occurs in Cushing's syndrome, causes central obesity, type 2 diabetes, and cardiovascular disease. In idiopathic obesity, circulating cortisol levels are essentially normal, although there may be a subtle impairment in the negative feedback by endogenous cortisol [8]. However, it has been suggested that changes in glucocorticoid metabolism may contribute to a Cushing's-like phenotype linked to abdominal obesity [6].

The availability of active glucocorticoid in different tissues is modulated through enzymatic interconversion between cortisone and cortisol by 11β-hydroxysteroid dehydrogenase type 1 and 2 (11βHSD1/2) [7]. 11βHSD1 activates cortisone to cortisol and is widely expressed with high expression in liver, adipose tissue and lung. 11βHSD2 perform the opposite reaction and is present in mineralocorticoid target tissues e.g. the kidney [7]. In idiopathic obesity, 11βHSD1 levels are increased in subcutaneous adipose tissue and reduced in liver [6], [9]. The physiological relevance of alterations in the activity of this enzyme has been demonstrated in several animal models. 11βHSD1 knockout mice are protected from visceral fat accumulation when fed a high-fat diet [10], while selective over-expression of 11βHSD1 in adipose tissue results in abdominal obesity, insulin resistance, and hypertension [11], [12]. Liver-specific over-expression of the enzyme results in a non-obese phenotype with increased hepatic fat content linked to mild insulin resistance, dyslipidemia, and hypertension [13].

Circulating cortisol levels are unaltered in post- versus premenopausal women [14]. Although not confirmed in humans [15], E2 attenuates 11βHSD1 expression and enzyme activity in the liver and testis of rodents, [16]–[18]. This suggest that the dysmetabolic features including fat redistribution, elevated blood pressure, and dyslipidemia commonly seen after menopause may at least partly be mediated through tissue-specific alterations in cortisol levels via increased 11βHSD1.

We hypothesized that 11βHSD1 expression and activity are increased in adipose tissue and/or liver in postmenopausal versus premenopausal normal weight women.

## Materials and Methods

### Subjects

Twenty-three premenopausal and 23 postmenopausal, healthy, normal weight women were recruited by advertisements in the local newspapers and within the Umeå University Hospital and campus areas. Exclusion criteria were: diabetes, thyroid dysfunction, hepatic and renal disease, use of tobacco, hormonal contraceptives, systemic gonadal hormone replacement therapy, or oral glucocorticoid medication. None of the postmenopausal women reported menstrual periods within the last 12 months. One premenopausal woman used inhaled steroids for asthma (budesonide, 400 µg/24 h). Three postmenopausal women had well-controlled hypertension treated with β-blockers, diuretics, or calcium antagonist, one took tolterodine for urinary incontinence and bisphosphonates for osteoporosis, and two used topical E2 or estriol treatment.

### Ethics Statement

The study was approved by the Umeå University Ethical Committee and all subjects gave written, informed consent before entering the study.

### Clinical protocol

Premenopausal women were evaluated during both follicular and luteal phases of the menstrual cycle to investigate the possible effects of hormonal fluctuations. Menstrual phase or postmenopausal status was confirmed by measuring serum E2 and progesterone levels. Anthropometric measurements, urine collections, adipose tissue biopsies, and hepatic cortisone conversion tests were performed on separate days.

Weight to the nearest 0.1 kg (with subjects wearing light clothes) and height and waist circumference to the nearest 0.5 cm was measured. Blood pressure was measured in the sitting position after 5 minutes rest with a mercury sphygmomanometer.

Urine was collected for 24 h, measured to the nearest ml, and aliquots stored without preservatives at −20°C until analyzed.

Approximately 2 g of periumbilical superficial subcutaneous adipose tissue was excised under local anaesthesia with lidocaine (Xylocaine® without adrenaline, AstraZeneca, Sweden) after an overnight fast. Tissue was snap frozen in liquid nitrogen within 5 minutes after removal, and stored at −80°C until further analyses.

For the hepatic cortisone conversion test, the subjects took an oral dose of 1 mg dexamethasone (2 tablets of 0.5 mg Decadrone®, Merck & Co., Sweden) at 2300 h in order to suppress endogenous cortisol production and fasted overnight. Venous blood samples for baseline cortisol levels were drawn at approximately 0800–0900 h, followed by 25 mg oral cortisone acetate (Cortal® 25 mg, N.V. Organon Oss Holland). Blood samples for serum cortisol analyses were then drawn every 30 minutes during the following four hours.

Venous blood samples for routine laboratory tests were drawn at the time of anthropometric measurements. Venous blood samples for serum analyses (described below) were drawn in the mornings of the adipose tissue biopsies and cortisone conversion tests after at least eight hours of fasting.

### Laboratory Methods

#### RNA extraction and quantification

Total RNA was extracted according to the manufacturer's instructions from approximately 450 mg of adipose tissue using the RNeasy® lipid tissue midi kit (Qiagen Nordic, Qiagen House, West Sussex, UK). RNA concentrations were measured on a ND-1000 Spectrophotometer (NanoDrop Technologies, Bancroft Building, Wilmington, DE, USA) and integrity was evaluated on a 1% agarose electrophoretic gel and visualized with ethidium bromide under UV-light.

SpectrophotometerTwo micrograms of RNA was reverse transcribed using TaqMan® Reverse Transcription Reagents (Roche Molecular Systems, Inc., Branchburg, NJ, USA). Real-time PCR was carried out on an ABI Prism® 7000 Sequence Detection System (Applied Biosystems) according to manufacturer's instructions using Universal PCR Master Mix 2X (Roche Molecular Systems, Inc., Branchburg, NJ, USA) and TaqMan Gene expression assays for target genes 11βHSD1 (assay No. Hs00194153_m1), aromatase (assay No. Hs00240671_m1), and the endogenous control Cyclophilin A (PPIA) (assay No. Hs99999904_m1) (Applied Biosystems, Foster City, CA, USA). All reactions were performed in triplicate and non-template controls were included on every plate. A standard curve was included on each plate for relative quantification. Data were normalized against PPIA, which had the lowest coefficient of variation and the best stability value, based on the Normfinder algorithm (http://www.mdl.dk/publicationsnormfinder.htm) out of three tested endogenous controls (PPIA, LRP10 and RPLP0, data not shown), previously found to be suitable for human adipose tissue analyses [19].

#### In vitro adipose 11βHSD1 enzyme activity assay

11βHSD1 protein in adipose tissue was quantified by measuring enzyme activity in the dehydrogenase direction, which is the preferred reaction in tissue homogenates with excess cofactor [20], [21]. Adipose tissue (300 mg) was homogenized in 900 µl ice-cold buffer (10% glycerol, 300 mM NaCl, 1 mM EDTA, 50 mM Tris, pH 7.4) and 0.1 mM dithiothreitol and centrifuged at 12,000×g for 15 min at 4°C. Total protein concentrations were determined using the Bradford technique (Bio-Rad protein assay, Bio-Rad Laboratories Inc., Herculus, CA, USA). Duplicate samples of 3 mg/ml of protein were incubated at 37°C with 10 mM NADP and 50 nM [1,2,6,7-^3^H]_4_-cortisol for 24 h. Samples were withdrawn at 12, 16, 20, and 24 h and frozen at −80°C. Subsequently, glucocorticoids were extracted with dichloromethane, the organic phase evaporated, extracts dissolved in ethanol and separated by thin layer chromatography (on TLC aluminium sheets, 20×20 cm, Silica gel 60 F_254_, Merck KGaA, Darmstadt, Germany, mobile phase; chloroform and ethanol (92∶8)). Radio-labelled glucocorticoids were detected by exposure of the TLC sheet to a tritium storage phosphor screen, subsequently scanned in a Typhoon™ 9400 scanner (both GE Healthcare Europe GmbH, Germany). 11βHSD1 activity was expressed as percent conversion of cortisol to cortisone.

#### Urinary corticosteroid metabolites

Cortisol, cortisone, 5α-tetrahydrocortisol (5α-THF), 5β-THF, and 5β-tetrahydrocortisone (THE) concentrations were analyzed by gas chromatography and electron impact mass spectrometry as previously described [22].

#### Serum analyses

Liver transaminases were measured in the samples drawn at the time of anthropometric measurements (menstrual phase not determined). Estradiol, progesterone and cortisol were measured in samples drawn both at the biopsy and at the cortisone conversion test. All other analyses were made in samples drawn on the morning of the adipose tissue biopsy to avoid interference with the dexamethasone suppression administered in advance of the cortisol conversion test. Serum cortisol, progesterone, sex hormone-binding globulin (SHBG), and insulin were analyzed by electrochemiluminescence immunoassays, on a Modular Analytics E170 (all from Roche AB, Stockholm, Sweden). Serum free testosterone was measured using a radioimmunoassay, Coat-a-count®, (Siemens Healthcare Diagnostics, Deerfield, IL, USA). Glucose, alanine aminotransferase (ALT), aspartate aminotransferase (AST), cholesterol, triglycerides, and HDL and LDL cholesterol were analyzed on a VITROS® Ektachem 950 IRC (Johnson & Johnson, Langhorne, PA, USA) using colorimetric assays. Apolipoprotein A1 (ApoA1) and Apo B was analyzed with immunoturbidimetric technique on a Hitachi 911 analyzer (Roche AB, Stockholm, Sweden). HOMA-IR (Homeostasis Model Assessment for Insulin Resistance) was calculated using the HOMA calculator v2.2 available at www.dtu.ox.ac.uk. E2 was measured using an ultra sensitive radioimmunoassay (ESTR-US-CT, CIS bio international, Gif-sur-Yvette, Cedex, France) (intra- and interassay coefficients of variation (CV); 2.8–18.1% and 5.8–17.6%, respectively).

### Statistical Analyses

Data are shown as mean±SD, unless otherwise indicated, and were natural log-transformed when necessary to achieve normal distribution. Student's t-tests were used to compare means between the groups and paired Student's t-tests to compare follicular and luteal phase samples in the premenopausal group. Associations between variables, including follicular phase premenopasual and postmenopausal women, were examined with Pearson correlation tests with adjustments for waist circumference, BMI, and menopausal status in partial correlation analyses. The effect of, and possible interaction between, waist circumference or BMI and menopausal status was tested with linear regression. Repeated-measures ANOVA was employed to test the effect of menopausal status on adipose enzyme activity. Statistical calculations were carried out using the SPSS software (release 14.0.1, SPSS Inc., 233 S. Wacker Drive, Chicago, IL). Premenopausal women found not to be in the anticipated menstrual phase were excluded in the statistical calculations for that particular test.

## Results

### Subject Characteristics (Tables 1 and 2)

BMI and waist circumference did not differ between the menopausal groups. E2, progesterone, and free testosterone levels were significantly lower in postmenopausal *vs.* premenopausal women regardless of menstrual phase. Systolic blood pressure, serum total cholesterol and cholesterol sub-fractions (except HDL; only *vs.* luteal phase), triglyceride levels, and liver transaminases were higher in postmenopausal women, while HOMA-IR was lower, mainly due to lower fasting insulin levels among postmenopausal women. Within the menstrual cycle, LDL, apolipoprotein B, and cholesterol levels were increased in the follicular *vs.* luteal phase.

**Table 1:** Subject characteristics.

	Premenopausal	Postmenopausal
*N*	*23*	*23*
Age (yrs)	27±5	63±4^***^
BMI (kg/m2)	23.3±1.8	23.4±1.9
Waist circumference (cm)	79.7±7.3	82.8±5.5
SBP (mmHg)	112±11	127±14^***^
DBP (mmHg)	69±9	74±9
AST (ukat/L)	0.32±0.06	0.41±0.07^***^
ALT (ukat/L)	0.31±0.09	0.43±0.07^***^
HOMA-IR	0.93±0.34	0.66±0.18**
Glucose (mmol/L)	4.62±0.35	4.72±0.40
Insulin (mIU/L)	7.3±2.4	5.1±1.7^***^

SBP, systolic blood pressure; DBP, diastolic blood pressure; AST, aspartate aminotransferase; ALT alanine aminotransferase; HOMA-IR, Homeostasis Model Assessment for Insulin Resistance.

** *P<*0.01 and ^***^P<0.001 *vs.* premenopausal women.

**Table 2:** Circulating levels of steroids and lipids and adipose aromatase transcript levels.

	Premenopausal (follicular phase)	Premenopausal (luteal phase)	Postmenopausal
*N*	*19*	*17*	*23*
*Circulating steroids*			
Estradiol (pmol/L)	200±142†	348±141	20±6^***†††^
Progesterone (nmol/L)	2.30±0.79^†††^	32.53±16.44	1.32±0.47^***†††^
Testosterone/SHBG	0.023±0.02	0.021±0.019	0.011±0.006* †
Cortisol (nmol/L)	476±184	738±207	472±141
*Blood lipids*			
Cholesterol (mmol/L)	4.1±0.8†	3.9±1.0	6.2±1.1^***†††^
HDL cholesterol (mmol/L)	1.66±0.39	1.54±0.38	1.83±0.31†
LDL cholesterol (mmol/L)	2.13±0.62^††^	2.08±0.72	3.91±1.02^***†††^
Triglycerides (mmol/L)	0.71±0.17	0.66±0.14	1.08±0.34^***†††^
ApoA1 (mg/L)	1,391±211	1,301±219	1,576±176^**†††^
ApoB (mg/L)	735±156^†††^	717±200	1,212±262^**†††^
*Adipose gene expression*			
Aromatase	11,203±7565	11,522±7454	11,863±5506

One woman in follicular phase and three in the luteal phase of the menstrual cycle did not have the biopsy and three women in each premenopausal group were found not to be in the correct menstrual phase. 11βHSD1, 11β-hydroxyteroid dehydrogenase type 1; PPIA, Cyclophilin A; SHBG, Sex hormone-binding globulin; HDL, high-density lipoprotein; LDL low-density lipoprotein; ApoA1, Apolipoprotein A-1; ApoB, Apolipoprotein B. N = 16 for luteal phase blood lipid data.

* *P<*0.05, ^**^ *P<*0.01 and^ ***^ *P<*0.001 *vs.* follicular phase.

† *P<*0.05, ^††^ *P<*0.01 and^ †††^ *P<*0.001 *vs.* luteal phase.

### Urinary Corticosteroid Metabolites (Table 3)

Total glucocorticoid urinary metabolite excretion did not differ between groups, indicative of unaltered glucocorticoid production rate with menopause. The (5α-THF+5β-THF)/THE ratio was significantly higher in postmenopausal *vs.* premenopausal women in luteal phase, indicating a higher total body 11βHSD1 activity in postmenopausal women; this difference remained after adjustment for BMI and waist circumference. The (5α-THF+5β-THF)/THE ratio correlated positively with diastolic blood pressure (R = 0.34, *P*<0.05) and serum LDL (R = 0.31, *P*<0.05) and negatively with serum E2 (R = −0.34, *P*<0.05); these associations did not remain after adjustment for BMI, waist circumference, and menopausal group (Table 4). Postmenopausal women had higher urinary 5β-THF excretion than premenopausal women regardless of menstrual phase, with lower 5α-THF/5β-THF ratio compared to premenopausal women in the follicular phase; these differences persisted after adjustment for waist circumference and BMI.

**Table 3:** Urinary corticosteroid metabolites.

	Premenopausal (follicular phase)	Premenopausal (luteal phase)	Postmenopausal
*N*	*18*	*18*	*23*
THE (µg/day)	2,480±1319	2,653±1988	2,507±698
5βTHF (µg/day)	1,010±522	986±643	1,586±432^***††^
5αTHF (µg/day)	1,117±750	1,023±886	975±427
Cortisone (µg/day)	116±36	110±45	113±34
Cortisol (µg/day)	117±49	93±42	91±32^*^
(5βTHF+5αTHF)/THE	0.96±0.59	0.87±0.47	1.08±0.28†
5αTHF/5βTHF	1.41±1.48	1.07±0.70	0.69±0.44^*^
5α-THF/cortisol	9.49±5.23	10.61±8.57	11.59±6.24
5β-THF/cortisol	9.17±5.17	11.59±7.68	19.69±8.18^***††^
THE/cortisone	21.36±9.79	23.16±13.29	24.12±11.43
Cortisol/cortisone	1.01±0.24	0.89±0.26	0.86±0.34^*^
Total urinary metabolites	4,840±2,341	4,865±3,193	5,273±1,002

Three women in each premenopausal group were found not to be in the anticipated phase of the menstrual cycle, one woman in follicular phase did not comply with the instructions of the urine collection and two women in luteal and one woman in follicular phase of the menstrual cycle did not collect urine. ^*^ *P*<0.05 and^ ***^ *P*<0.001 *vs.* follicular phase.

† *P*<0.05 and ^††^ *P*<0.01 *vs.* luteal phase. THF, tetrahydrocortisol; THE, tetrahydrocortisone.

**Table 4:** Bivariate correlations for adipose 11βHSD1 expression and activity, urinary THFs/THE and hepatic 11βHSD1 activity versus anthropometric data, sex steroids, hormones and gene expression of aromatase.

	Correlation coefficients (Pearson correlation)			
	Adipose 11βHSD1mRNA	Adipose 11βHSD1-EA	Urinary THFs/THE	Hepatic 11βHSD1-EA
*N*	*42*	*33*	*41*	*40*
11βHSD-EA	0.85^***^	n.a.	n.a.	n.a.
*Anthropometric variables*				
Waist circumference	0.56^***^	0.66^***^	0.31	−0.01
BMI	0.10	0.25	0.07	−0.15
SBP	0.44^**^	0.30	0.27	**0.38** *
DBP	0.22	0.17	**0.34** *	0.16
*Blood analyses*				
Insulin	−0.13	0.07	−0.17	−0.09
Glucose	0.04	0.07	−0.08	−0.01
Cholesterol	**0.39** *	0.20	0.29	0.58^***,^ ‡
HDL cholesterol	**−0.35** *, ‡	−0.48^**,^ ‡	−0.01	0.18
LDL cholesterol	0.50^**^	**0.35** *	**0.31** *	0.58^***,^ ‡
Triglycerides	**0.34** *	0.21	0.17	0.29
*Sex steroids*				
Aromatase mRNA	0.58^***,^ ‡	**0.44** *	n.a.	n.a.
Estradiol	−0.30	−0.16	**−0.34** *	−0.48^**^
Progesterone	−0.21	−0.09	−0.20	−0.08
Testosterone/SHBG	0.02	0.10	−0.18	**−0.33** *

Premenopausal women in follicular phase of the menstrual cycle and postmenopausal women were included. Enzyme activity was measured as percent conversion of cortisol to cortisone after 24 h incubation. Urinary THFs/THE gives a measure of whole body 11βHSD1 activity. Hepatic 11βHSD1 activity was measured as serum cortisol 30 minutes after oral cortisone. 11βHSD1, 11β-hydroxysteroid dehydrogenase type 1; EA, Enzyme activity; THFs/THE, (5α-tetrahydrocortisol+5β-tetrahydrocortisol)/5β-tetrahydrocortione; SBP, systolic blood pressure; DBP, diastolic blood pressure, n.a., not applicable. Data are correlation coefficients (Pearson correlation).

* *P<*0.05, ^**^ *P<*0.01, and^ ***^ *P<*0.001.

‡ Association significant after adjusting for waist circumference, BMI, and menopausal group (partial correlation).

### 11βHSD1 in Subcutaneous Adipose Tissue

**Figure 1:**
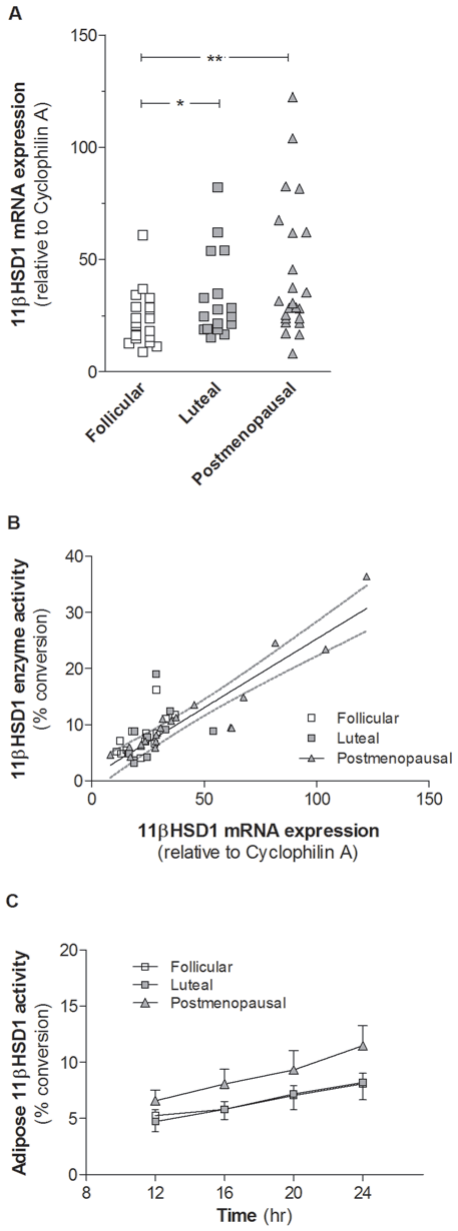
Subcutaneous adipose tissue 11βHSD1. **A** 11βHSD1 transcript levels were normalized to endogenous control Cyclophilin A. *P<0.05, ** *P*<0.01, N = 19, 17, and 23 for the premenopausal follicular (□), luteal (▪), and postmenopausal (▴) groups, respectively. One woman in follicular phase and three in the luteal phase of the menstrual cycle did not have the biopsy and three women in each premenopausal group were found not to be in the correct phase. Data were natural log-transformed to achieve normal distribution. **B** Correlation between adipose 11βHSD1 activity after 24 hr incubation (percent conversion) and adipose 11βHSD1 mRNA expression (relative to Cyclophilin A). Premenopausal follicular (□), luteal (▪), and postmenopausal women (▴). Linear regression line is shown for the postmenopausal group, dotted lines denotes the 95% confidence interval. **C** Subcutaneous adipose tissue 11βHSD1 activity was measured as percent conversion of cortisol to cortisone over time in tissue homogenates, protein concentration 3 mg/ml. N = 13, 10, and 20 for premenopausal follicular (□), luteal (▪), and postmenopausal women (▴), respectively. There were no significant differences between the groups. Data were ln-transformed to achieve normal distribution and are shown as means±SEM.

Among premenopausal women 11βHSD1 expression was higher in luteal *vs*. follicular phase of the menstrual cycle (Fig. 1A). Postmenopausal women had a higher adipose 11βHSD1 expression *vs.* follicular phase premenopausal women. 11βHSD1 expression in adipose tissue correlated with waist circumference (R = 0.56, *P*<0.001) but not BMI (Table 4). Linear regression analysis showed that waist circumference and menopausal group both independently affect the 11βHSD1 expression in adipose tissue; *β* = 0.493, *P*<0.001 and *β* = −0.298, *P*<0.05, respectively.

11βHSD1 enzyme activity showed a close correlation with 11βHSD1 mRNA expression (Fig. 1B) but did not differ significantly between groups (Fig. 1C). Enzyme activity was positively associated with waist circumference (R = 0.66, *P*<0.001), this remained after adjustment for BMI and menopausal group (Table 4).

11βHSD1 expression correlated positively with systolic blood pressure, cholesterol, LDL cholesterol, triglycerides, and adipose aromatase expression (Table 4). In addition there was a negative correlation with HDL cholesterol (Table 4). Of these correlations, only the associations with aromatase and HDL cholesterol remained after adjustment for BMI, waist circumference, and menopausal group. A similar pattern was shown for adipose 11βHSD1 activity which correlated positively with LDL cholesterol and aromatase and negatively with HDL cholesterol (Table 4); only the latter remained after adjustments.

### In Vivo Hepatic 11βHSD1 Activity

**Figure 2:**
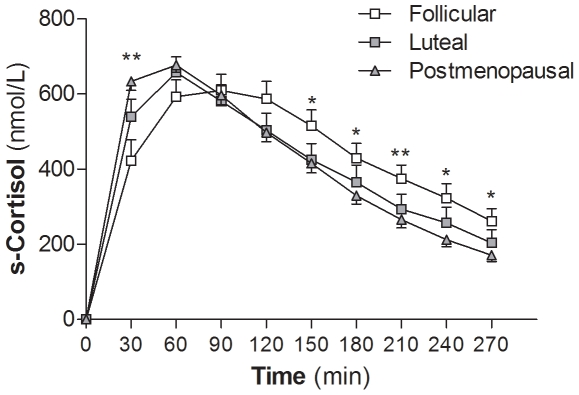
Hepatic 11βHSD1 activity. Serum cortisol levels after overnight dexamethasone suppression and oral cortisone intake (25 mg). Postmenopausal women (▴, N = 23) had higher serum cortisol levels at 30 min post cortisone intake than premenopausal women in follicular phase of the menstrual cycle (□, N = 17). *P<0.05 and **P<0.01 for postmenopausal *vs.* premenopausal follicular phase. Luteal phase, ▪, N = 16. Data are means±SEM.

At 30 minutes post oral cortisone ingestion, serum cortisol levels were higher in postmenopausal women than premenopausal women in the follicular phase of the menstrual cycle (*P*<0.01, Fig. 2); the difference remained after adjustment for BMI and waist circumference. This indicates a faster conversion of cortisone to cortisol on first pass through the liver, suggesting increased hepatic 11βHSD1 reductase activity in the postmenopausal women.

Similar to the measures of adipose 11βHSD1, serum cortisol levels at 30 min post cortisone ingestion, correlated positively with systolic blood pressure, serum cholesterol, and LDL cholesterol. The associations to serum cholesterol and LDL cholesterol remained after adjustment for BMI, waist circumference, and menopausal group (Table 4). There were also negative correlations with serum estradiol and testosterone levels before adjustments (Table 4).

## Discussion

The postmenopausal women in this study had a higher urinary cortisol/cortisone metabolites ratio suggestive of increased total body 11βHSD1 activity. Importantly, we found higher transcript levels of the glucocorticoid-generating enzyme 11βHSD1 in subcutaneous fat with concomitantly increased first-pass conversion of cortisone to cortisol, suggesting increased hepatic 11βHSD1 activity. These findings support an elevation in 11βHSD1 activity in older menopausal women in both adipose tissue and liver, resulting in a tissue-specific increase in glucocorticoid exposure despite unaltered circulating cortisol levels. Waist circumference was closely linked with increased adipose tissue 11βHSD1 transcript levels and enzyme activity. However, 11βHSD1 transcript levels in adipose tissue were also independently affected by menopausal/age group. Additionally, the differences in hepatic and whole-body 11βHSD1 activity between premenopausal and postmenopausal women were independent of waist circumference, suggesting an effect of age/menopausal group over and above the associations between centralization of body fat and 11βHSD1.

Whether these changes are related to age and/or menopause per se cannot be determined from this cross-sectional study. Notably, decreased circulating estrogen levels due to ovariectomy or menopause in rodents and/or humans results in increased body fat mass with central distribution [23], [24]. This change in body composition, linked to insulin resistance and increased risk of cardiovascular disease in postmenopausal women, can be reversed by estrogen replacement therapy [23], [24]. However, data from studies of the effects of estrogen on 11βHSD1 in adipose tissue are sparse and partly conflicting, and included mixed cohorts of both pre- and postmenopausal women, with varying BMI, and/or a limited number of samples [25]–[27]. In our study serum E2 levels correlated negatively with measures of both whole body and hepatic 11βHSD1 activity, supporting a down-regulatory effect of E2. However, this is confounded by putative effects of age/menopausal status and body composition. Notably, we did not find an association between serum E2 levels and adipose 11βHSD1 expression or activity, which may suggest tissue-specific differences in the interaction between estrogen and 11βHSD1.

In contrast, we found a positive association between aromatase and 11βHSD1 gene expression in adipose tissue, independent of adiposity and menopausal group. In postmenopausal women, aromatase activity is the main source of E2, and aromatase expression has been shown to increase after menopause [25], [28]. The positive correlation with aromatase hence suggests an up-regulatory effect of E2 on 11βHSD1 expression in adipose tissue, but this may also be related to glucocorticoids driving the promoter activity of the aromatase gene [29]. Therefore, our observations regarding E2 and 11βHSD1 are inconsistent and need further investigation to elucidate whether estrogen deficiency underpins elevated 11βHSD1 in the postmenopausal state.

There are alterations in cytokines and other hormones with age and menopause which may influence 11βHSD1 levels [30]. With menopause there is an increased immune activation with increased serum levels of proinflammatory cytokines [31]. Interestingly, cytokines such as TNF-α influence 11βHSD1 expression and activity [32], and it would therefore be of interest to study possible associations between immune responses and glucocorticoid activation in these subjects. An alternative explanation related to menopausal status is progesterone deficiency, which has not been studied in detail. Notably, we did not find any associations between serum progesterone levels and measures of 11βHSD1 expression/activity. However, among premenopausal women adipose 11βHSD1 expression was higher in the luteal phase of the menstrual cycle, which is characterized by high serum estrogen and progesterone levels, compared to the follicular phase. On the other hand, consistent with previous studies [15] we did not find differences in urinary steroid ratios, or liver 11βHSD1 activity within the menstrual cycle. Further experimental analyses of the role of progesterone in tissue-specific glucocorticoid activation therefore seem warranted.

11βHSD1 activity and mRNA expression in adipose tissue were strongly correlated but there was no significant difference in 11βHSD1 activity between groups. The lack of a statistically significant difference may at least partly be due to low power as there were fewer samples available for these analyses due to lack of tissue homogenate. Importantly, 11βHSD1 activity is relatively low in this BMI range (compared to levels seen in obese subjects, [21]) and in line with this, the previously reported association between 11βHSD1 activity/expression and BMI [9], [21] was not detected in this study cohort.

Our results from the hepatic cortisone-to-cortisol conversion test indicate higher 11βHSD1 activity in the liver of postmenopausal women. This contrast with previous observations in overweight individuals, where we and others have suggested a reduced 11βHSD1 activity in liver in combination with up-regulation of activity/expression in adipose tissue [9], [20], [21]. Interestingly, gonadal dysfunction and increased body fat percentage with normal body weight is linked to increased hepatic cortisone conversion in patients with myotonic dystrophy [33]. These patients develop fatty livers and, although liver fat content was not estimated in the present study, the postmenopausal women of our study had significantly higher serum levels of liver enzymes (AST, ALT), which is linked to increased liver fat content.

The balance between cortisol and cortisone and their metabolites in urine has often been used as an estimation of the whole body activity of 11βHSD1. We found a higher THFs/THE ratio in postmenopausal women, which is in part explained by their higher 5βTHF levels. This is consistent with an overall increase in 11βHSD1 activity with menopause/older age, and similar to what we have previously observed with increasing BMI in pre-/perimenopausal women, but contrary to the findings in a parallel study in men [9], [21]. However, we did not find an association between the THFs/THE ratio and BMI and we only saw a trend towards a positive correlation with waist circumference. Notably, the urinary THFs/THE ratio does not distinguish the activities of 11βHSD type 1 and 2, the latter which catalyses the deactivation of cortisol to cortisone. Moreover, the changes in 5α-THF/5β-THF ratio and of 5β-THF/cortisol ratio in postmenopausal women raise the possibility of confounding effects due to variation in 5β-reductase activity. Increased 5β-reductase would be expected to decrease, rather than increase, the plasma cortisol values after oral cortisone. With the hepatic cortisone-to-cortisol conversion-test data in mind we therefore conclude that there is increased liver 11βHSD1 activity after the menopause but this may not be the only reason for alterations in urinary cortisol metabolite ratios. There is thus a need for further studies using isotopically labelled cortisol to evaluate whole body 11βHSD1 enzyme activity [34].

Unfortunately we did not have the opportunity to assess visceral adipose tissue expression and activity of 11βHSD1. This would have been most interesting especially with regard to the redistribution of adipose tissue commonly seen with menopause with increased abdominal depots. Although animal studies indicate higher 11βHSD1 levels in the visceral adipose tissue, at least with obesity, some human studies have shown higher expression in the subcutaneous depot [20], [35]–[41].

In accordance with previous studies in transgenic mice [11]–[13] we found that, overall, higher 11βHSD1 was associated with higher blood pressure and a less beneficial blood lipid profile; higher total cholesterol, low-density lipoprotein, and triglycerides and lower high-density lipoprotein, which links to increased risk of cardiovascular disease. However, we did not find any associations with insulin or glucose. Rather, the lower HOMA-IR indices found in postmenopausal women was mainly due to lower fasting insulin levels in this group. Interestingly, insulin secretion is lower in postmenopausal vs. premenopausal women and this may contribute to our finding [42]. However, this observation may also be explained by genetic factors, diet and/or exercise habits, since the older women have managed to remain lean past the menopause. It should thus be considered that by recruiting normal weight postmenopausal women we may have introduced a bias in this cohort.

In conclusion, we suggest that menopause/older age linked to central fat distribution in normal weight women is associated with increased tissue-specific glucocorticoid exposure via 11βHSD1. This may contribute to dysmetabolic changes linked to the increased risk of cardiovascular disease in postmenopausal women.
